# Enhancing resistance to *Salmonella typhimurium* in yellow-feathered broilers: a study of a strain of *Lactiplantibacillus plantarum* as probiotic feed additives

**DOI:** 10.3389/fmicb.2024.1450690

**Published:** 2024-11-20

**Authors:** Yangyan Yin, Hao Peng, Huili Bai, Zhe Pei, Zhongwei Chen, Chunxia Ma, Min Zhu, Jun Li, Changting Li, Yu Gong, Leping Wang, Ling Teng, Zhongsheng Qin, Jianhui Zhou, Tianchao Wei, Yuying Liao

**Affiliations:** ^1^Institute of Animal Science and Technology, Guangxi University, Nanning, China; ^2^Key Laboratory of Veterinary Biotechnology, Guangxi Veterinary Research Institute, Nanning, China; ^3^Key Laboratory of China (Guangxi)-ASEAN Cross-Border Animal Disease Prevention and Control, Ministry of Agriculture and Rural Affairs of China, Nanning, China; ^4^Virginia Polytechnic Institute and State University, Blacksburg, VA, United States; ^5^Guizhou Provincial Livestock and Poultry Genetic Resources Management Station, Guiyang, China; ^6^Guilin Animal Epidemic Disease Prevention and Control Center, Guilin, China

**Keywords:** probiotics, yellow-feathered broiler, growth performance, intestine tissue, gut health, immune response

## Abstract

*Lactiplantibacillus plantarum* strains are potentially rich sources of probiotics that could help avoid infections. In order to evaluate their efficacy in bolstering resistance to *Salmonella typhimurium* infection among chicks. In this study, *L. plantarum* and commercial probiotics were administered via the water supply at a dosage of 1×10^9^ CFU per chicken from days 1 to 7 to establish a protective system for the chicks. On days 8 and 9, *S. typhimurium* was attacked to investigate the preventive effects and potential mechanisms of *L. plantarum* in comparison with commercial probiotics. Post-treatment, we took a broad range of measurements, including body weight, immune organ index changes, the viable count of *S. typhimurium* in the liver, spleen, and cecum, as well as pathological changes in the liver. Our findings demonstrated that both *L. plantarum* and the commercial probiotic could safeguard chicks from *S. typhimurium* infection. The data also suggested that probiotic medication could ease weight loss postinfection, lower the bacterial count in the liver, spleen, and cecum, and attenuate liver pathological damage among all treated participants. Subsequently, we did high-throughput sequencing of 16S rRNA to examine the fecal microbiota of the chicks 5  days post-infection. We discovered that both *L. plantarum* and the commercial probiotic could fend off the invasion of *S. typhimurium* by affecting the bacterial population of *Anaerotruncus, Colidextribacter*, and *Lactobacillus*. Generally speaking, the addition of *L. plantarum* as a feed additive protects yellow-feathered broilers from *S. typhimurium* illness, suggesting great potential for commercial uses in the poultry industry.

## 1 Introduction

In poultry production, chicks are reared in a distinct, isolated environment that minimally interacts with the external world. Contrary to other mammals, newly hatched chicks do not inherit maternal antibodies; however, they do acquire a beneficial microbial population from adult chickens through daily interactions. The intestinal microbiota of these newly hatched chicks is rudimentary, displaying low diversity and density (Apajalahti et al., 2004; Javadi et al., 2024; Jiangrang et al., 2003). Consequently, their intestinal colonization predominantly arises from environmental sources and is fragile when exposed to pathogens. Studies have reported that *Salmonella* can readily colonize and interact with the intestinal epithelium of newly hatched chicks (Dragana et al., 2013).

*Salmonella*, a Gram-negative facultative anaerobic bacterium from the Enterobacteriaceae family, is a primary contributor to various poultry diseases. It leads to substantial economic losses due to growth retardation and increased mortality rates within the poultry industry. As one of the major foodborne pathogens, it also poses a significant food safety risk globally (Eng et al., 2015; Ferrari et al., 2019; Norma and Santos, 2018). At present, over 2,600 serotypes of *Salmonella* exist, with numerous pathogens affecting both chickens and humans (Santos et al., 2018). It has been reported that a *Salmonella* infection can significantly hinder broiler growth (Abudabos et al., 2014), causing symptoms like mucosal damage, diarrhea, and reduced feed intake (Vandeplas et al., 2009). Due to the immature immune system of young chickens, *Salmonella* infections are frequent (Abudabos et al., 2018) and can be easily transmitted to humans through the consumption of poultry meat, leading to severe gastrointestinal diseases (Wilson et al., 2016).

With the global demand for poultry meat on the rise (Assem et al., 2021; Maeve et al., 2017), over 65 billion chickens are reared each year for protein production, with an anticipated increase of 16% by 2030 (OECD, Food and Agriculture Organization of the United Nations, 2016). Antibiotics have been employed in the poultry industry to thwart *Salmonella* infection among young chickens (Wenguang et al., 2018). Unfortunately, this preventive method has led to a grave food safety concern: antibiotic resistance. Both China and the European Union enacted bans on the use of growth-promoting antibiotics as feed additives in 2020 and 2006, respectively (Guo et al., 2020). Hence, the quest for alternative poultry feed additives has emerged as an urgent necessity for both food safety and global public health (Celi et al., 2017). In addition, the declining efficacy of antibiotics against bacterial pathogens such as *Salmonella*, combined with the rise of antibiotic-resistant bacteria, underscores the need for novel approaches to prevent or treat infections caused by intestinal pathogens.

At present, a range of methods have proven effective in preventing *Salmonella* infection, particularly the utilization of probiotics (Abudabos et al., 2018; Wilson et al., 2016; Khan and Naz, 2013). Probiotics are living microorganisms that, when consumed or applied, are intended to bestow health benefits. The earliest probiotics primarily consisted of a single microorganism, typically from the *Saccharomyces* or *Lactobacillus* genera. Benefits of probiotics include competing with pathogens for intestinal receptor sites, producing specific metabolites (such as short-chain fatty acids, hydrogen peroxide, and other antimicrobial substances), and eliciting immunostimulatory effects (Madsen et al., 2001). These protective mechanisms assist in inhibiting and eliminating potential pathogens such as *Salmonella*, improving the intestinal microenvironment, fortifying the intestinal barrier, reducing inflammation, and enhancing certain antigen-specific immune responses (Lin and Zhang, 2017; Markowiak and Śliżewska, 2017). *Lactiplantibacillus plantarum* (*L. plantarum*), a common strain of lactic acid bacteria, exhibits probiotic characteristics. In this study, *L. plantarum* was isolated from the intestine of a healthy chicken. This strain, functioning as a probiotic, demonstrated robust growth under simulated gastrointestinal conditions and exhibited strong antibacterial effects against both Gram-negative and Gram-positive bacteria. Additionally, it possessed the capability to degrade corn gibberellin and aflatoxin (Yin et al., 2023). Therefore, this study progresses to employ this specific strain of *L. plantarum* in investigating the resistance of chicks to *Salmonella typhimurium* (*S. typhimurium*) infection, while also exploring its probiotic properties in depth. Multiple studies have demonstrated that it benefits the growth performance of broilers (Jinfeng et al., 2018) and aids in the prevention and control of diseases (Baikui et al., 2021).

Over the past 70 years, researchers have primarily focused on improving poultry through genetic enhancement and innovative feed compositions. However, adequate attention might not have been directed towards controlling microbial infections such as *Staphylococcus*, *Salmonella*, *Streptococcus*, and *Campylobacter* (Agyare et al., 2019). Broiler chickens are especially susceptible to bacterial infections in the initial few weeks when their immune systems are still developing (Ohimain and Ofongo, 2012). Furthermore, it can take up to 8 weeks to establish a stable intestinal microbiota (Lutful, 2009). The longer it takes to achieve bacterial homeostasis, the higher the risk of pathogenic bacterial infection (Irshad, 2006). In this study, one-day-old broiler chickens were utilized to evaluate the protective effects against *Salmonella* when either *L. plantarum* or a commercial probiotic was added to their drinking water. Results indicated that the preventive addition of *L. plantarum* and the commercial probiotic could alleviate weight loss induced by *Salmonella* infection, reduce the *Salmonella* count in the liver, spleen, and cecum, and lessen liver pathological damage. Additionally, probiotics could further prevent *Salmonella* infection by modulating the intestinal microbiota in young chicks.

## 2 Materials and methods

### 2.1 Bacteria strains and testing animals

All animal procedures in this study adhered to the protocol approved by the Institutional Animal Ethics Examination Committee of the Guangxi Veterinary Research Institute in Nanning, China (Approval No. 8/2014/JU). The probiotics used in the study included *L. plantarum* GX17, obtained from the Key Laboratory of Biotechnology at the Guangxi Veterinary Research Institute. The strain was isolated from the intestinal contents of healthy chickens. The specific steps are as follows: the intestinal contents were gradiently diluted using PBS, 100 μL of the diluted liquid was spread on MRS solid culture medium for culture to obtain single colonies, and Gram staining was performed after multiple purifications to observe the staining results and morphological characteristics. The species of the isolated bacteria was preliminarily identified as *Lactobacillus* according to Bergey’s manual of systematic bacteriology, 2nd edition (Boone et al., 2001). The bacterial genomic DNA was extracted, and PCR amplification and sequencing of the 16S rDNA sequence were performed. For more accurate identification, we determined its whole genome sequence, and the result was the same as 16S rDNA sequencing, so the strain was determined to be *L. plantarum*. The whole genome information of *L. plantarum* GX17 is available in the NCBI Sequence-Read-Archive repository [CP159198, CP159199, CP159200, CP159201, CP159202]. The commercial probiotic product contains probiotic, composed of chicken-derived *Lactobacillus*, *Streptococcus thermophilus*, their metabolites, and glucose. It was sourced from Shandong Baolai Lilai Biotechnology Co., Ltd. The pathogenic bacterium used in the study was *S. typhimurium* (strain SM022), generously provided by Professor Alejandro Aballay from Duke University, USA. One-day-old healthy yellow-feathered broiler chickens were purchased from Guangxi Jinling Agriculture and Animal Husbandry Group Co., Ltd.

### 2.2 Experimental designs

**Figure 1:**
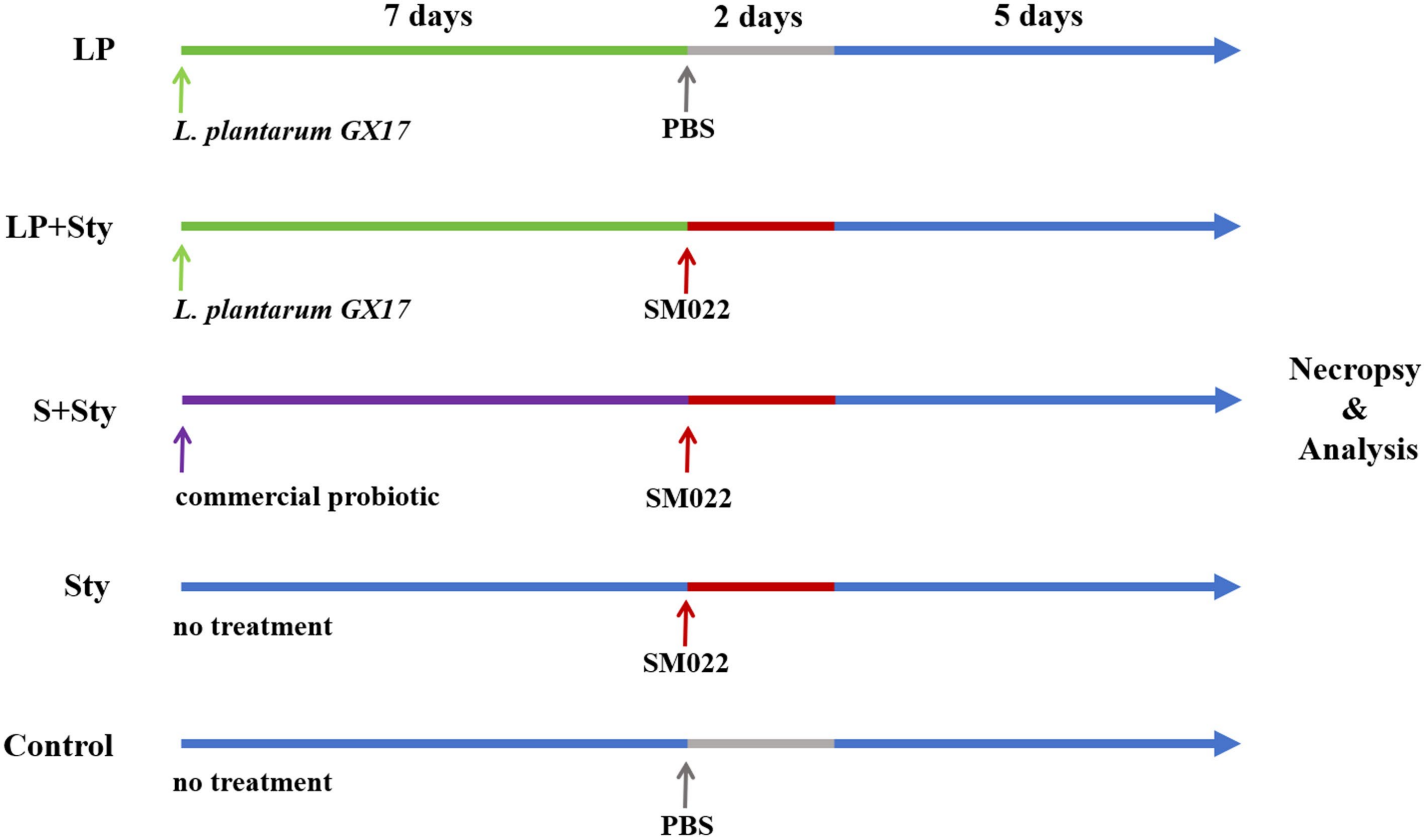
Experimental design for chick treatment groups. *n* = 75 each group. Control group: chicks were orally administered PBS on the 8th and 9th days. LP group: chicks were orally administered *L. plantarum* from the 1st to the 7th day and given PBS on the 8th and 9th days. LP + Sty group: chicks were orally administered *L. plantarum* from the 1st to the 7th day and were given *S. typhimurium* on the 8th and 9th days. S + Sty group: chicks were orally administered a commercial probiotic from the 1st to the 7th day and given PBS on the 8th and 9th days. Sty group: chicks were orally administered *S. typhimurium* on the 8th and 9th days.

A total of 375 one-day-old yellow-feathered chicks were randomly divided into 5 groups (with 5 replicates per group and 15 chicks per replicate): the *L. plantarum group* (LP group), the *L. plantarum* + *S. typhimurium* group (LP + Sty group), the commercial probiotic + *S. typhimurium* group (S + Sty group), the *S. typhimurium* group (Sty group), and the control group (no disposed). The average initial body weight did not significantly vary among the groups. In the LP group, during the first 7-day period, chicks were provided with drinking water supplemented with 1.5 × 10^9^ colony-forming units (CFU) of live *L. plantarum* per chick. On days 8 and 9, they were orally administered 1 mL phosphate buffered saline (PBS) and had free access to food and water for the rest of the time. In the LP + Sty experimental group, during the first 7-day period, chicks drank water supplemented with 1.5 × 10^9^ CFU of live *L. plantarum* per chick daily. On days 8 and 9, each chick was orally administered 1 mL of *S. typhimurium* at a concentration of 1.5 × 10^9^ CFU/mL. In the S + Sty group, during the first 7-day period, chicks were provided with drinking water supplemented with 1.5 × 10^9^ CFU of a commercial probiotic per chick daily. On days 8 and 9, each chick was orally administered 1 mL of *S. typhimurium* at a concentration of 1.5 × 10^9^ CFU/mL. In the Sty group, on days 8 and 9 of the experiment, each chick was orally administered 1 mL of *S. typhimurium* at a concentration of 1.5 × 10^9^ CFU/mL, while for the rest of the time, they had free access to feed and water. The control group orally administered 1 mL PBS during days 8 and 9 of the experiment, and for the rest of the time, they had free access to feed and water. Before feeding the probiotics, water was withheld for 1 h each day. The probiotic solution was consumed within 3 h, after which the drinking containers were completely washed and then refilled with regular drinking water. The control group received PBS during days 8–9 of the experiment, and for the rest of the time, they had free access to feed and water. The experimental design is shown in Figure 1.

### 2.3 The duration of testing cycles

The study was conducted at the experimental base of the Guangxi Veterinary Research Institute. Prior to initiating the experiment, the chicken house was meticulously cleaned and disinfected. The chickens were housed in cages under natural lighting conditions, with unrestricted access to feed and water. Regular observations were made and records kept of the chicken’s feeding behavior and overall health status. The experiment spanned 14 days, with relevant measurements taken on the 14th (final) day.

The initial ambient temperature for the chicks’ cultivation was maintained at 31°C, with a gradual reduction of 2°C per week. The feed utilized in this experiment was formulated based on the “Chicken Feeding Standards” (NY/T 33-2004), taking into consideration the growth cycle and specific traits of the yellow-feathered broilers. The composition and nutritional content of the basal diet are presented in Table 1.

**Table 1:** Composition of basal diets (air-dry basis).

Items	Percentage (%)	Nutrient levels	Percentage (%)
Corn	55.00	ME (MJ/kg)^b^	12.45
Soybean meal	36.20	CP	21.15
Soybean oil	3.90	Ca	0.92
Limestone	1.15	P	0.65
NaCl	0.25	Lysine	1.18
CaHPO_4_	1.95	Methionine	0.47
Premix^a^	1.55		
Total	100.00		

a The premix for 1 to 21 days of ages provided the following per kg of diets: VA 15,000 IU, VD 35,100 IU, VE 19.2 IU, VK3 2.4 mg, VB1 1.2 mg, VB2 10.2 mg, VB6 2.4 mg, VB12 0.012 mg, D-pantothenic acid 12 mg, nicotinic acid 39 mg, folic acid 1.2 mg, biotin 0.189 mg, choline 700 mg, Gu (as copper sulfate) 8 mg, Mn (as manganese sulfate) 100 mg, Fe (as ferrous sulfate) 80 mg, Zn (as zinc sulfate) 60 mg, I (as potassium iodide) 0.35 mg, Se (as sodium selenite) 0.15 mg.

b ME is calculated value. Other nutrient levels are measured values.

### 2.4 Chicken weight changes

The weight of each group of chicks was measured on the morning of the 7th and 14th days at 9:00 a.m. on an empty stomach. The weight changes before and after the infection were recorded for each group.

### 2.5 Immune organ index

On the 5th day after infection (5 dpi), three chicks from each group were euthanized. Their thymus, spleen, and bursa of Fabricius were collected aseptically. Adhering fat tissues were dissected, and the weight of each organ was measured to calculate the organ index. Immune organ index = immune organ weight (g)/chicken weight (kg).

### 2.6 Impact on the quantity of *Salmonella typhimurium* in chicken liver/spleen/ceca

On the 5 dpi, aseptic collection of chicken liver, spleen, and cecum was performed. Each organ was placed in sterilized Eppendorf tubes separately, weighed, and then suspended in sterile PBS buffer at a ratio of 1 g/mL. After thorough grinding and homogenization, the suspensions were serially diluted with PBS buffer. A volume of 100 μL of the diluted liquid was plated onto Xylose Lysine Desoxycholate agar plates and incubated at 37°C for 16 to 24 h. Colonies were counted to calculate the number of viable bacteria per gram of tissue.

### 2.7 Tissue pathological examination

On the 5 dpi, three chickens from each group were euthanized for tissue collection. Chicken liver, spleen, duodenum, and ceca were collected aseptically. The tissue was fixed in a 4% formaldehyde solution for 48 h, then sent to the Guangxi University of Chinese Medicine for further preparation.

### 2.8 Intestinal microbiota analysis

On the 5 dpi, respectively, three chickens from each testing group were randomly selected and sacrificed after overnight fasting to collect cecal contents. Total genomic DNA was extracted using the Bacterial Genome DNA Extraction Kit (Kangwei Century Biotechnology Co., Ltd., Beijing, China) following the manufacturer’s instructions. DNA concentration and integrity were measured with an Implen NanoPhotometer and agarose gel electrophoresis. The extracted DNA was stored at −20°C until further processing. The extracted DNA was used as a template for polymerase chain reaction (PCR) amplification of bacterial 16S ribosomal RNA (rRNA) genes with barcoded primers and Takara Ex Taq (Takara Bio Inc., Kyoto, Japan). For bacterial diversity analysis, V3–V4 variable regions of 16S rRNA genes were amplified with universal primers 343F (5′-TACGGRAGGCAGCAG-3′) and 798R (5′-AGGGTATCTAATCCT-3′) for V3–V4 regions (Herlemann et al., 2011).

The amplicon quality was visualized using agarose gel electrophoresis. The PCR products were purified with AMPure XP beads (Beckman Coulter, Inc., Pasadena, CA, United States) and amplified for another round of PCR. After being purified with the AMPure XP beads again, the final amplicon was quantified using the Qubit dsDNA Assay Kit (Thermo Fisher Scientific, Waltham, MA, United States). The concentrations were then adjusted for sequencing. Sequencing was performed on an Illumina NovaSeq 6000 with 250 bp paired-end reads (Illumina Inc., San Diego, CA).

The library sequencing and data processing were conducted by OE Biotech Co., Ltd. (Shanghai, China). Raw data was collected and stored in FASTQ format. The Cutadapt software was used to trim off the primer sequences. For the qualified paired-end raw data, an open-source software package DADA2 (version 2020.11) (Callahan et al., 2016) with the default settings of QIIME 2 (version 2020.11) (Bolyen et al., 2019) was employed to de-noise, removing sequencing errors, and merge and remove chimera sequences. The representative sequences and amplicon sequence variants (ASV) were then generated. After selecting representative sequences for each ASV using the QIIME 2 package, all representative sequences were annotated against the Silva (version 138) database. The species annotation was performed using the q2-feature-classifier software (version 2020.11) with default parameters. The α and β diversity analyses were then carried out using QIIME 2 software. The alpha diversity of samples was assessed using indices including Chao1 (Chao and Bunge, 2015) and Shannon (Hill et al., 2002). Unweighted UniFrac distance matrices were calculated by R software (version 4.2.2) and then used for unweighted UniFrac principal coordinates analysis (PCoA) to evaluate the β diversity of samples. ANOVA for differential gene expression analysis was running in R software.

### 2.9 Statistical analysis

The experimental data were statistically analyzed using SPSS 25.0. Data were presented as means and pooled as the standard error of the mean (SEM). The effects of dietary treatment on the measured variables were analyzed by one-way ANOVA, followed by Duncan’s multiple comparison test to compare among groups. Differences were considered significant at *p* < 0.05.

## 3 Results

### 3.1 The body weight changes among groups of treatments

**Figure 2:**
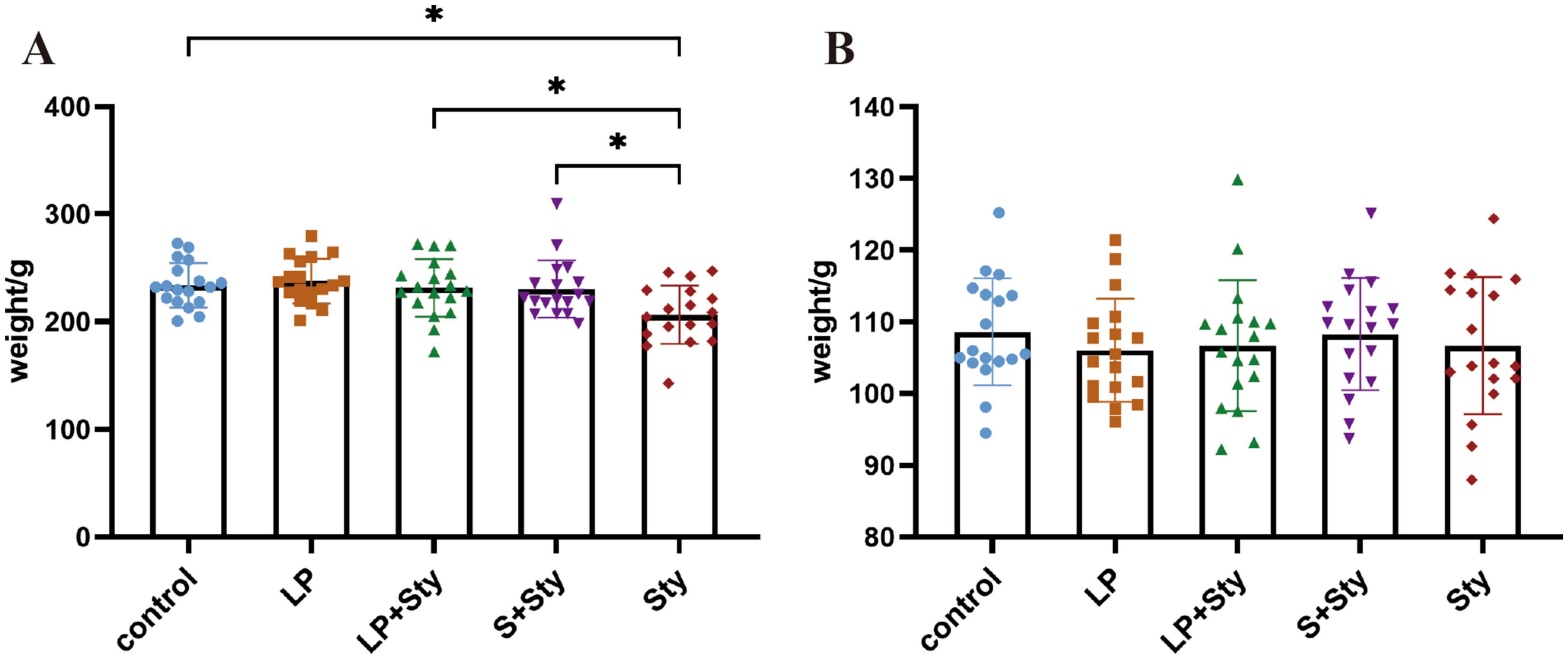
Body weight changes of chicks before and after *S. typhimurium* attack (^*^p < 0.05). (A) On the day of *S. typhimurium* attack. (B) 5 dpi. Control group: chicks were orally administered PBS on the 8th and 9th days. LP group: chicks were orally administered *L. plantarum* from the 1st to the 7th day and given PBS on the 8th and 9th days. LP + Sty group: chicks were orally administered *L. plantarum* from the 1st to the 7th day and were given *S. typhimurium* on the 8th and 9th days. S + Sty group: chicks were orally administered a commercial probiotic from the 1st to the 7th day and given pbs on the 8th and 9th days. sty group: chicks were orally administered *S. typhimurium* on the 8th and 9th days.

As indicated in Figure 2, by the day of infection, animals had been receiving probiotics in their drinking water for 7 days, except for Sty and control group. We observed no significant differences in body weight among the experimental groups (*p* > 0.05). On the 5 dpi, compared to the control group, the average body weight of the Sty group significantly decreased (*p* < 0.05). Meanwhile, the LP, LP + Sty, and S + Sty groups exhibited no notable variation (*p* > 0.05). When compared to the LP + Sty and S + Sty groups, the Sty group exhibited a significant decrease in body weight (*p* < 0.05). Specifically, the average body weight reduction in the Sty group was 24.49 g, 24.85 g, and 23.92 g relative to the control, LP + Sty, and S + Sty groups, respectively. These results indicate that probiotics can mitigate weight loss after the infection of *S. typhimurium*.

### 3.2 All immune organ indexes do not change among groups

**Figure 3:**
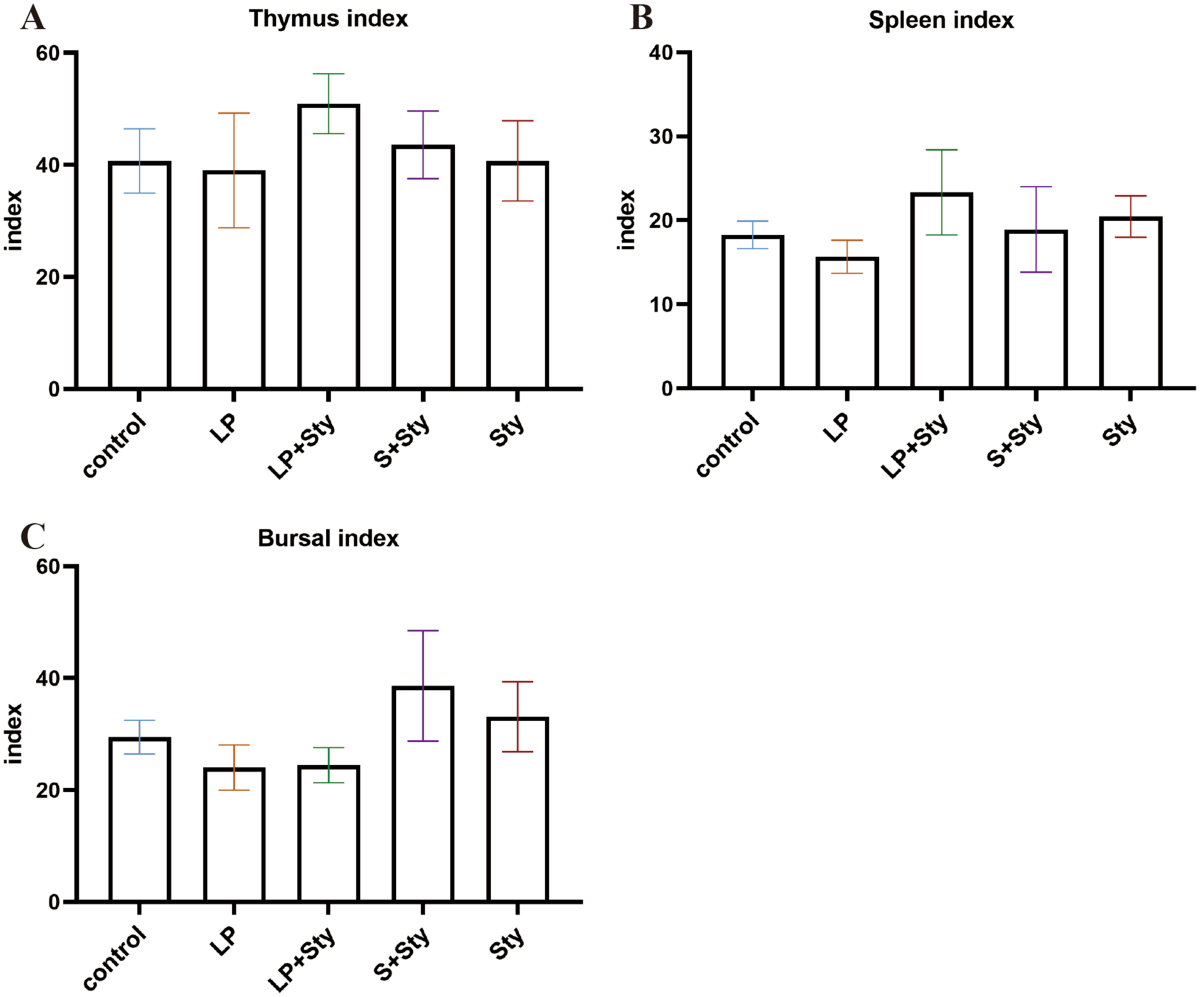
Comparison of immune organ indexes in different groups (A) Thymus index. (B) Spleen index. (C) Bursa of Fabricius index. Immune organ index = immune organ weight (g)/chicken weight (kg). Control group: chicks were orally administered PBS on the 8th and 9th days. LP group: chicks were orally administered *L. plantarum* from the 1st to the 7th day and given PBS on the 8th and 9th days. LP + Sty group: chicks were orally administered *L. plantarum* from the 1st to the 7th day and were given *S. typhimurium* on the 8th and 9th days. S + Sty group: chicks were orally administered a commercial probiotic from the 1st to the 7th day and given PBS on the 8th and 9th days. Sty group: chicks were orally administered *S. typhimurium* on the 8th and 9th days.

We also tested the change in immune organ index and presented all the data in Figure 3. Compared to the control group, there were no significant differences in the immune organ weights of the thymus, spleen, and bursa of Fabricius among groups (*p* > 0.05).

### 3.3 *Lactiplantibacillus plantarum* GX17 reduces the quantity of *Salmonella typhimurium* in the liver, spleen, and cecum of infected chicks

**Figure 4:**
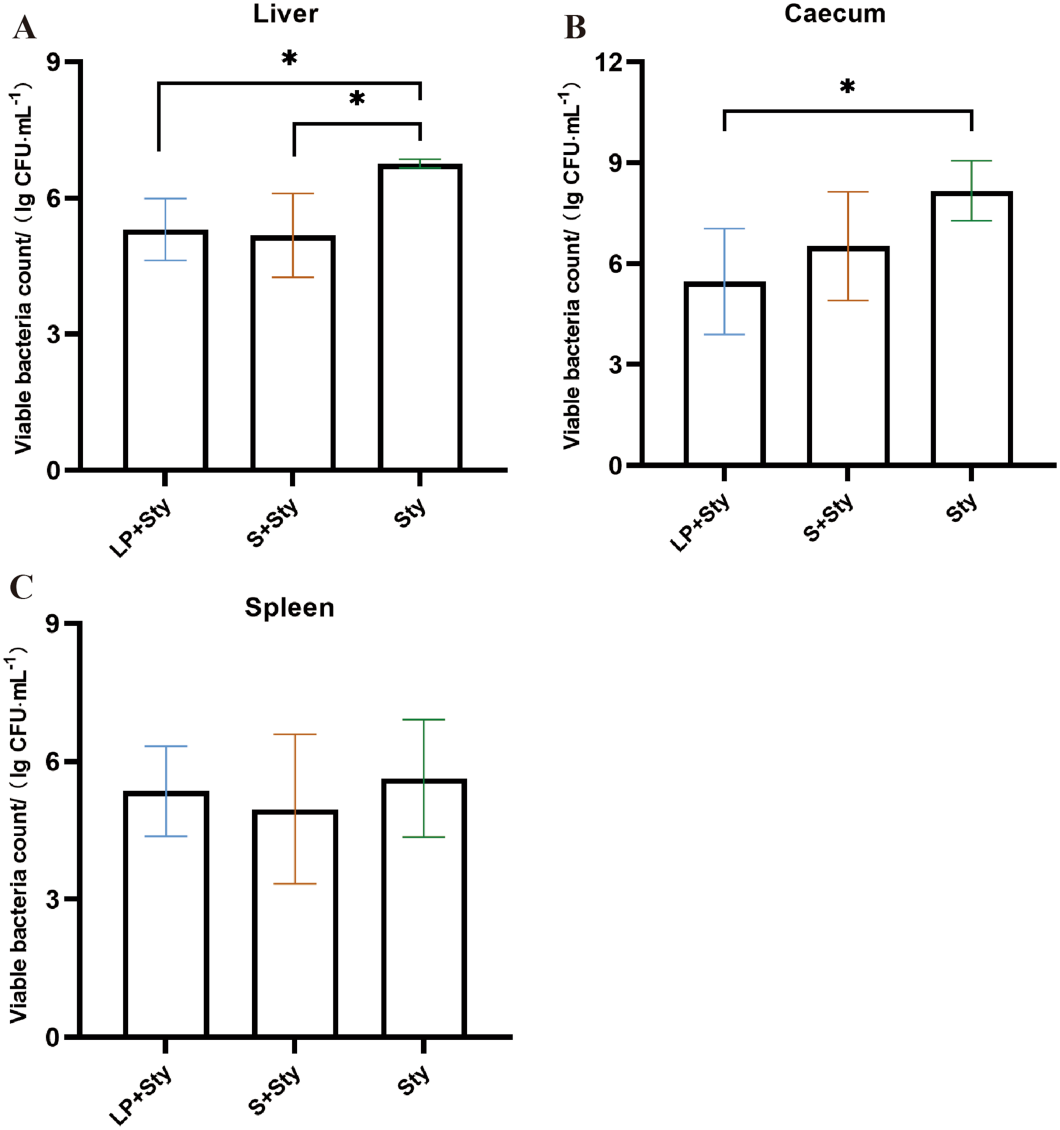
Determination of bacterial load in liver, spleen and cecum tissues of chicks in each group (^*^p < 0.05, ^**^p < 0.01, and ^***^p < 0.001). (A) Liver. (B) Spleen. (C) Cecum. Control group: chicks were orally administered PBS on the 8th and 9th days. LP group: chicks were orally administered *L. plantarum* from the 1st to the 7th day and given PBS on the 8th and 9th days. LP + Sty group: chicks were orally administered *L. plantarum* from the 1st to the 7th day and were given *S. typhimurium* on the 8th and 9th days. S + Sty group: chicks were orally administered a commercial probiotic from the 1st to the 7th day and given PBS on the 8th and 9th days. Sty group: chicks were orally administered *S. typhimurium* on the 8th and 9th days.

Five days post-infection, we conducted viable bacterial counts on the liver, spleen, and cecal contents of chicks. As illustrated in Figure 4, the quantities of *S. typhimurium* in the liver, spleen, and cecal contents of the LP + Sty and S + Sty groups were significantly lower compared to the Sty group (*p* < 0.05). Specifically, the concentration of *S. typhimurium* in the liver exceeded 10^6^ CFU/g in the Sty group, marking a level 12.23 times and 12.36 times higher than that in the LP + Sty group and S + Sty group, respectively. In the spleen, *S. typhimurium* concentrations surpassed 10^6^ CFU/g in the Sty group, which was 12.65 times and 13.38 times greater than the LP + Sty group and S + Sty group, respectively. In the cecum, *S. typhimurium* quantities exceeded 10^8^ CFU/g in the Sty group, which was 11.92 times and 11.72 times the counts in the LP + Sty group and S + Sty group, respectively. These findings suggest that both *L. plantarum* and the commercial probiotic can significantly reduce the quantity of *S. typhimurium* in the liver, spleen, and cecum of chicks (*p* < 0.05).

### 3.4 *Lactiplantibacillus plantarum* GX17 mitigates the histopathological changes in infected chicks

**Figure 5:**
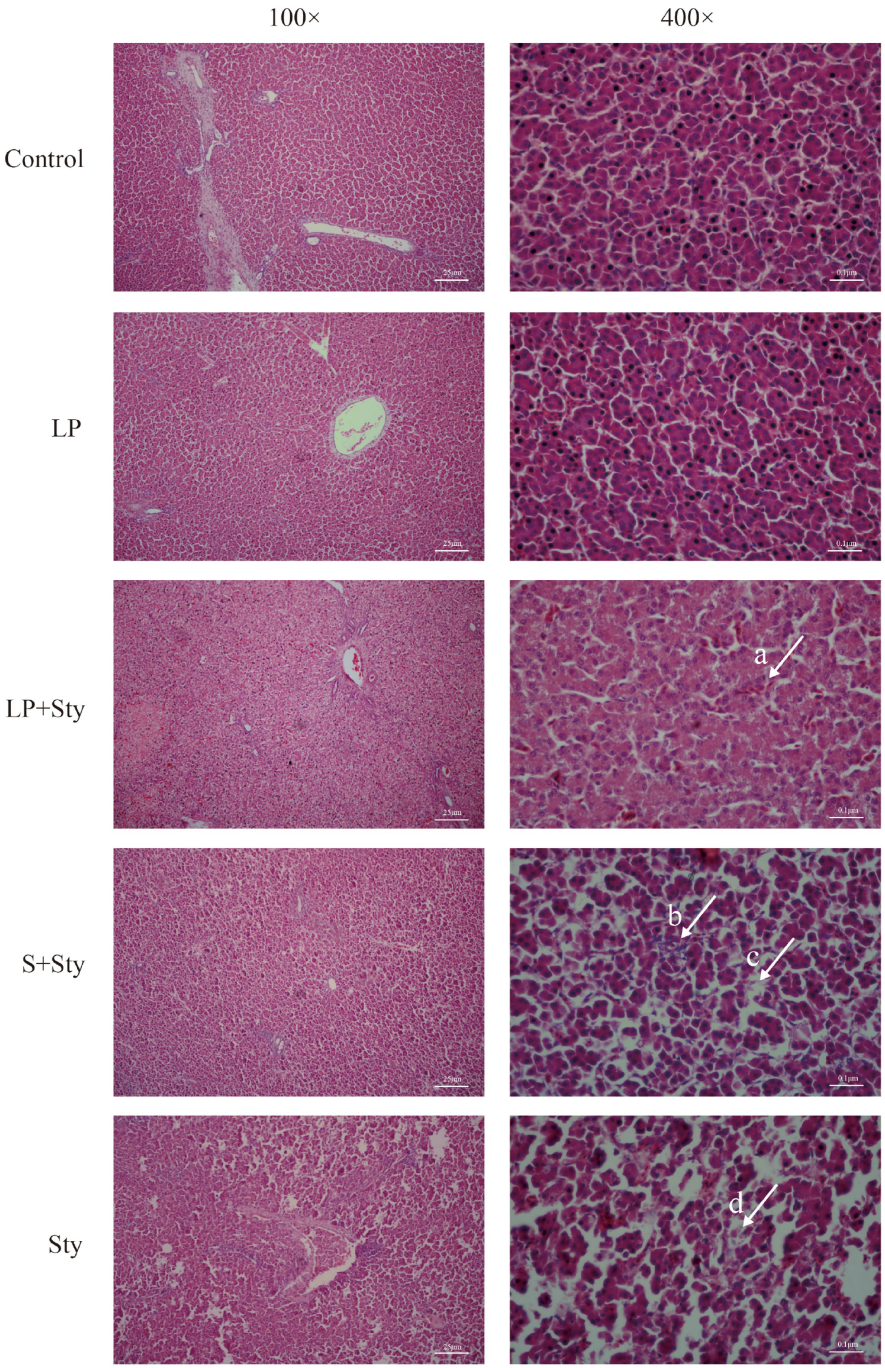
H&E staining results of chick liver tissues. (Arrow a) Filled with a few red blood cells. (Arrow b) Severe inflammatory cell infiltration. (Arrow c) The structure of hepatocytes is blurred. (Arrow d) Hepatocyte necrosis. Control group: chicks were orally administered PBS on the 8th and 9th days. LP group: chicks were orally administered *L. plantarum* from the 1st to the 7th day and given PBS on the 8th and 9th days. LP + Sty group: chicks were orally administered *L. plantarum* from the 1st to the 7th day and were given *S. typhimurium* on the 8th and 9th days. S + Sty group: chicks were orally administered a commercial probiotic from the 1st to the 7th day and given PBS on the 8th and 9th days. Sty group: chicks were orally administered *S. typhimurium* on the 8th and 9th days.

Upon examining the liver paraffin sections of chicks (Figure 5), it is apparent that the liver structure of the LP group and control group chicks is well-defined and intact. All liver cells in this study are neatly and regularly arranged, with a distribution radiating outward from the central vein. In the Sty group (infected with *S. typhimurium* only), the liver tissue exhibits a blurry structure, with congestion in the blood vessels, disordered arrangement of liver cells, infiltration of red blood cells, extensive damage to cell membranes, nuclear condensation, significant hepatocyte necrosis, and severe infiltration of inflammatory cells. In the LP + Sty and S + Sty groups, the liver tissue maintains a relatively intact structure with neatly arranged liver cells compared with the Sty group. There is mild congestion, with a small amount of inflammatory cells and slight necrosis, significantly alleviating the severity of liver lesions compared to the Sty group. These experimental results indicate that *L. plantarum* GX17 and the commercial probiotic have a mitigating effect on liver pathology following *S. typhimurium* infection.

### 3.5 The α and β diversity of cecal microbiota after *Salmonella typhimurium* infection

The 16S rRNA sequencing generated millions of raw reads. The sample coverage indexes for all 15 samples were above 99%. Good coverage (Supplementary Figure S1A), sparsity (Supplementary Figure S1B), Shannon-Wiener index (Supplementary Figure S1C), and ASV species accumulation curve (Supplementary Figure S1D) of all samples indicate sufficient data sampling and sequencing depth, covering almost all microbial communities in the 16S rRNA database.

**Figure 6:**
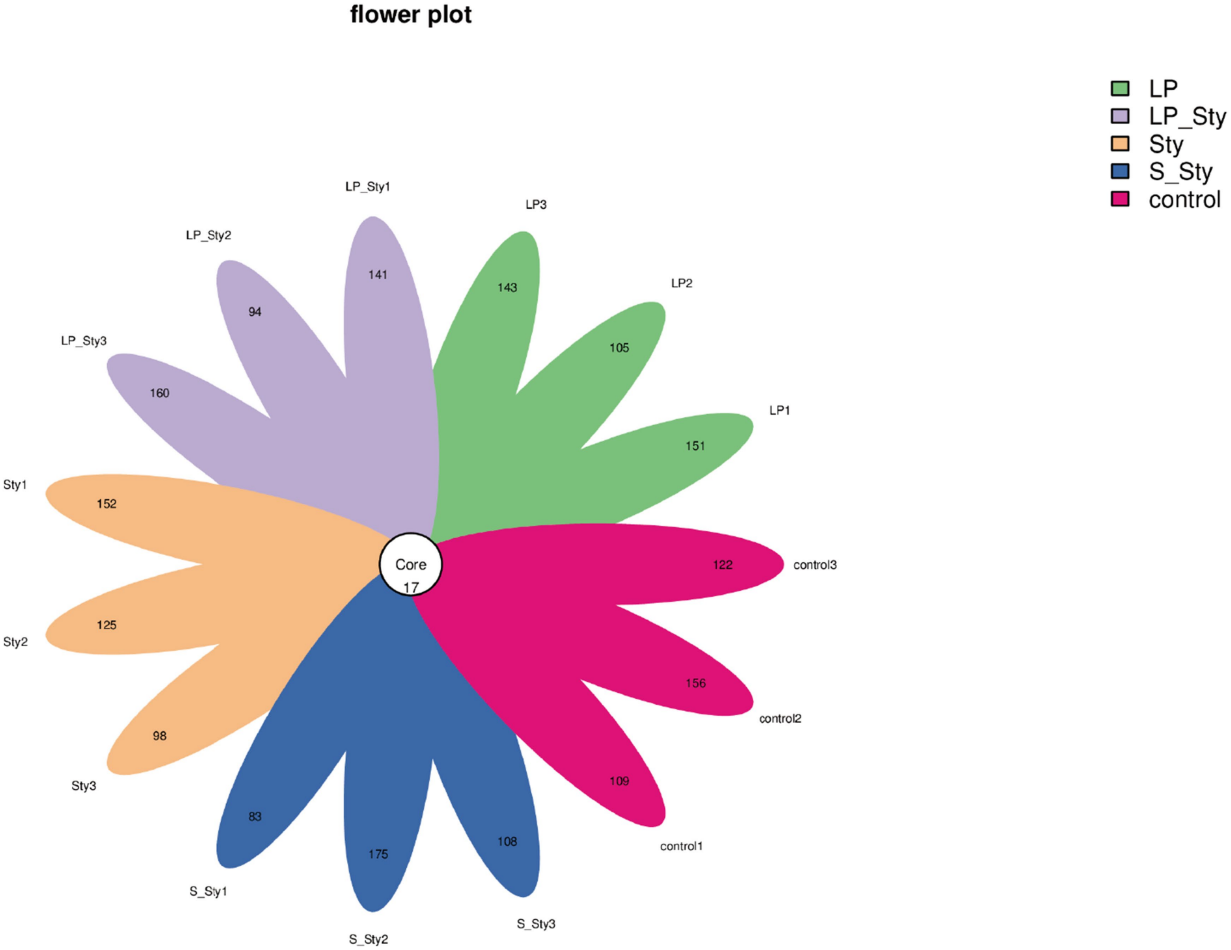
Flower plot of amplicon sequence variants (ASVs). This figure presents a flower plot illustrating the distribution of amplicon sequence variants (ASVs) across different samples. Each petal represents a unique ASV, with the length of the petal corresponding to the relative abundance of the ASV within the sample. The central circle denotes the core ASVs shared among all samples. Control group: chicks were orally administered PBS on the 8th and 9th days. LP group: chicks were orally administered *L. plantarum* from the 1st to the 7th day and given PBS on the 8th and 9th days. LP + Sty group: chicks were orally administered *L. plantarum* from the 1st to the 7th day and were given *S. typhimurium* on the 8th and 9th days. S + Sty group: chicks were orally administered a commercial probiotic from the 1st to the 7th day and given PBS on the 8th and 9th days. Sty group: chicks were orally administered *S. typhimurium* on the 8th and 9th days.

A total of 1,939 ASVs were obtained in this study. The number of ASVs in the LP, LP + Sty, Sty, S + Sty, and control groups were 450, 446, 426, 417, and 438, respectively. These results show that the fecal microbiota composition of the LP group was the highest, while the S + Sty group had the lowest, but the difference was not statistically significant. There were 17 ASVs shared among all five groups (Figure 6).

**Figure 7:**
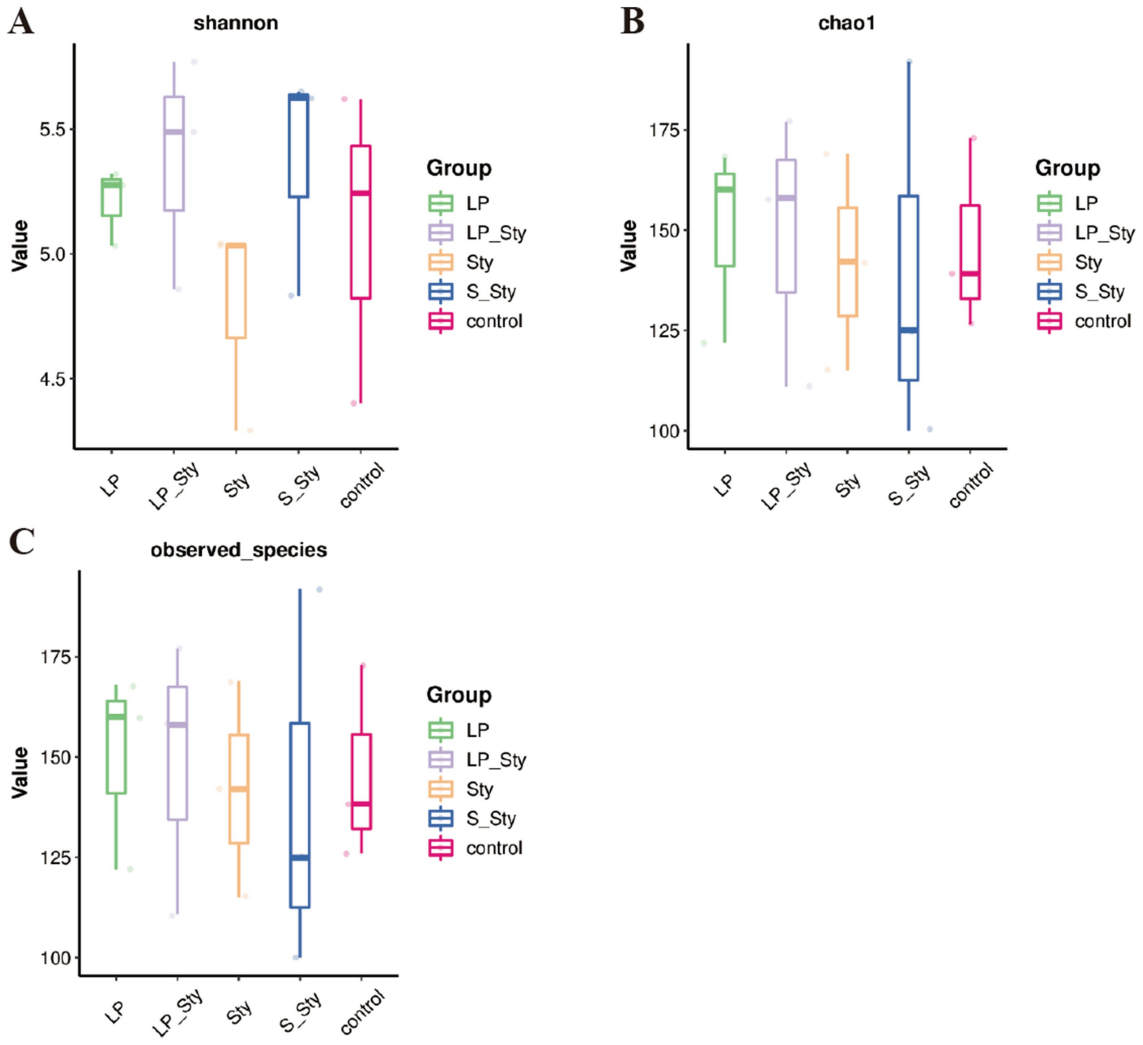
Alpha diversity indices. (A) Chao1—estimates species richness, accounting for rare species. (B) Observed species—reflects the actual number of distinct species observed. (C) Shannon—incorporates both species richness and evenness, providing a comprehensive measure of diversity. Control group: chicks were orally administered PBS on the 8th and 9th days. LP group: chicks were orally administered *L. plantarum* from the 1st to the 7th day and given PBS on the 8th and 9th days. LP + Sty group: chicks were orally administered *L. plantarum* from the 1st to the 7th day and were given *S. typhimurium* on the 8th and 9th days. S + Sty group: chicks were orally administered a commercial probiotic from the 1st to the 7th day and given PBS on the 8th and 9th days. Sty group: chicks were orally administered *S. typhimurium* on the 8th and 9th days.

**Figure 8:**
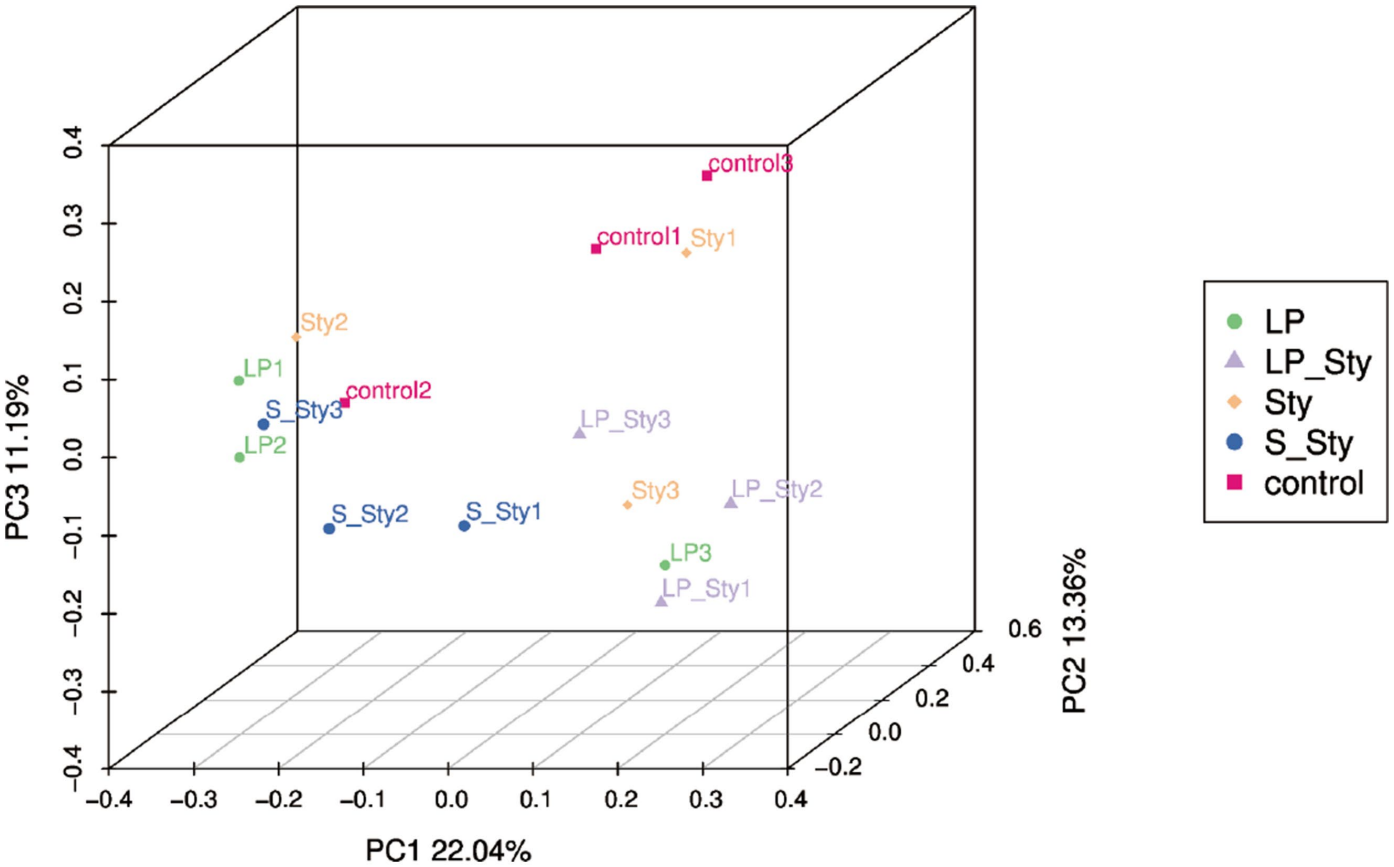
Principal coordinates analysis of microbial community composition. Control group: chicks were orally administered PBS on the 8th and 9th days. LP group: chicks were orally administered *L. plantarum* from the 1st to the 7th day and given PBS on the 8th and 9th days. LP + Sty group: chicks were orally administered *L. plantarum* from the 1st to the 7th day and were given *S. typhimurium* on the 8th and 9th days. S + Sty group: chicks were orally administered a commercial probiotic from the 1st to the 7th day and given PBS on the 8th and 9th days. Sty group: chicks were orally administered *S. typhimurium* on the 8th and 9th days.

The Chao1 species richness estimator (Figure 7A) and observed species index (Figure 7B) were used to measure the richness of microbial species, whereas the Shannon diversity index (Figure 7C) measures their diversity. Both indexes were calculated to evaluate the *α* diversity in our data. There were no significant differences in alpha diversity indices among groups. PCoA showed significant differences in beta diversity among the five groups. Adonis statistical analysis yielded a *p*-value of 0.021 (see Figure 8).

### 3.6 Bacterial classification in the cecum of chicks

**Figure 9:**
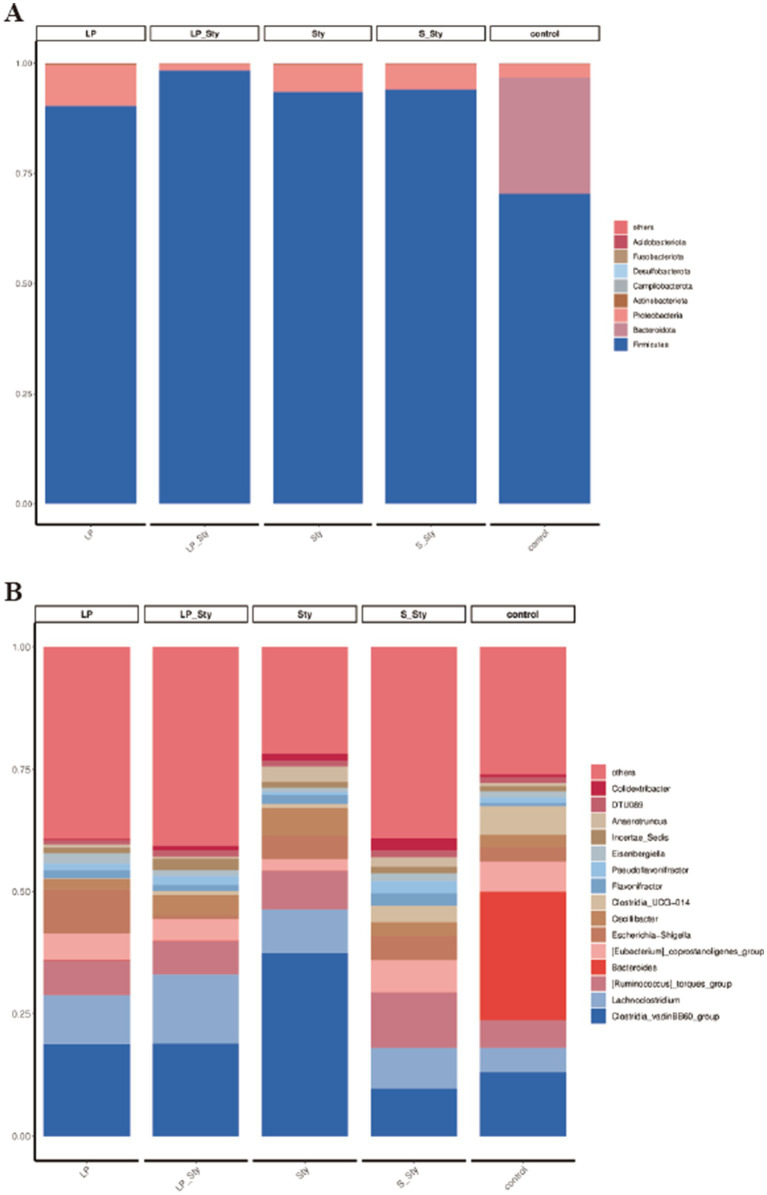
Comparative analysis of microbial community diversity. (A) Phylum level. (B) Genus level. Control group: chicks were orally administered PBS on the 8th and 9th days. LP group: chicks were orally administered *L. plantarum* from the 1st to the 7th day and given PBS on the 8th and 9th days. LP + Sty group: chicks were orally administered *L. plantarum* from the 1st to the 7th day and were given *S. typhimurium* on the 8th and 9th days. S + Sty group: chicks were orally administered a commercial probiotic from the 1st to the 7th day and given PBS on the 8th and 9th days. Sty group: chicks were orally administered *S. typhimurium* on the 8th and 9th days.

To elucidate the effects of *S. typhimurium* infection and probiotic supplementation on the composition of the cecal microbiota, we analyzed the bacteria at the phylum and genus levels to characterize the dynamics of microbial distribution. At the phylum level, except for the control group, where Firmicutes and Bacteroidetes were dominant, accounting for 70.37% and 26.33%, respectively, the cecal microbiota in the other groups were primarily composed of Firmicutes and Proteobacteria. Compared to the control group, the other groups had higher proportions of Proteobacteria and lower proportions of Bacteroidetes (Figure 9A). We further compared the bacterial composition in the cecum at the genus level. The relative abundance of the *Clostridia* genus was 18.83, 18.91, 37.48, 9.71, and 13.05% in the LP, LP + Sty, Sty, S + Sty, and control groups, respectively. These data suggest a significant *Clostridia* enrichment during *S. typhimurium* infection (Figure 9B).

**Figure 10:**
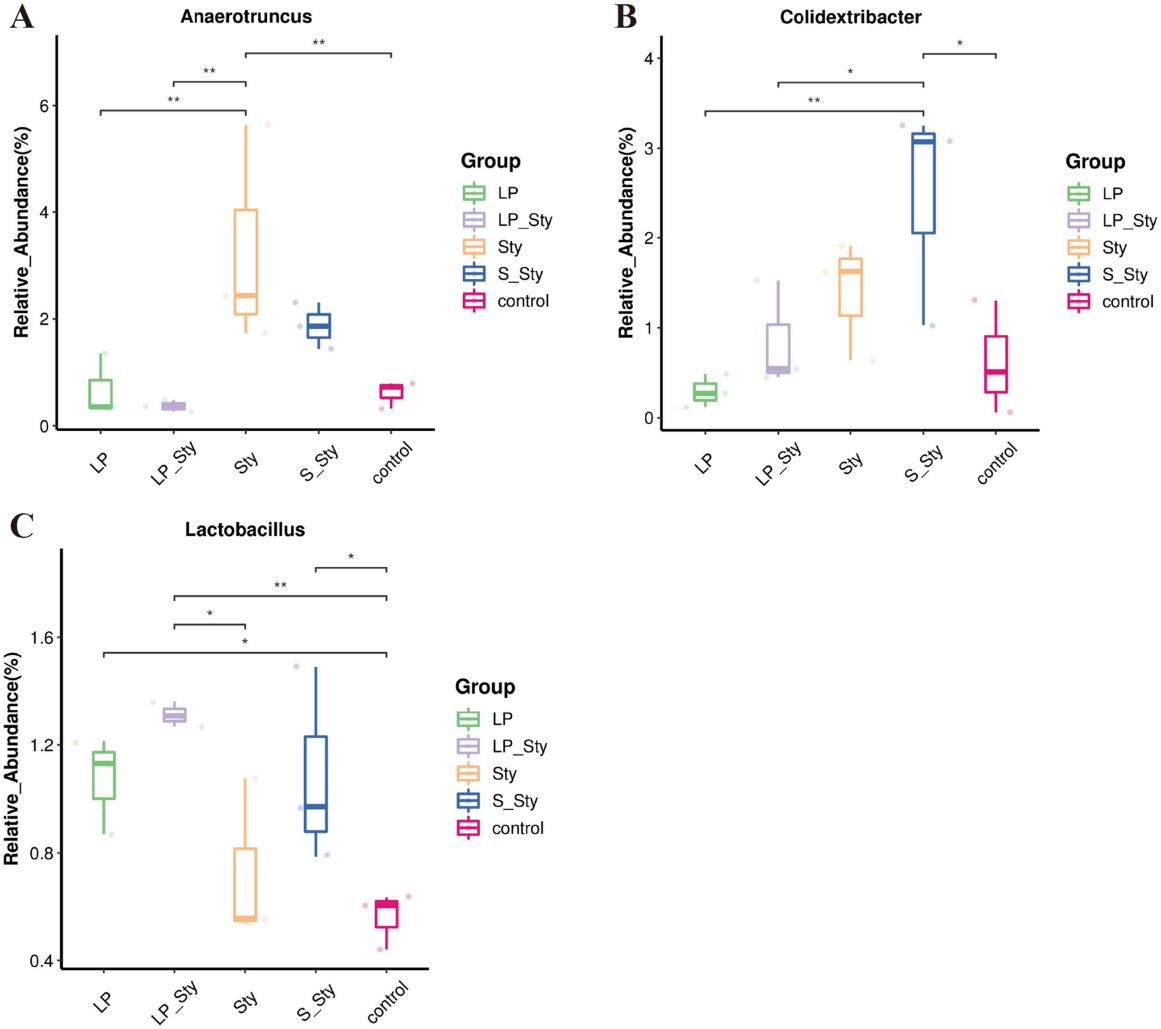
Boxplot analysis of genus-level species abundance (^*^p < 0.05 and ^**^p < 0.01). (A) *Anaerotruncus*. (B) *Colidextribacter*. (C) *Lactobacillus*. This figure presents a boxplot analysis to compare the species abundance of select genera within the microbial community. Control group: chicks were orally administered PBS on the 8th and 9th days. LP group: chicks were orally administered *L. plantarum* from the 1st to the 7th day and given PBS on the 8th and 9th days. LP + Sty group: chicks were orally administered *L. plantarum* from the 1st to the 7th day and were given *S. typhimurium* on the 8th and 9th days. S + Sty group: chicks were orally administered a commercial probiotic from the 1st to the 7th day and given PBS on the 8th and 9th days. Sty group: chicks were orally administered *S. typhimurium* on the 8th and 9th days.

ANOVA analysis (Figure 10) revealed that the relative abundance of the *Anaerotruncus* genus was significantly higher in the Sty group compared to the LP, LP + Sty, and control groups, indicating that *S. typhimurium* infection significantly increased the relative abundance of *Anaerotruncus* (*p* < 0.05). The relative abundance of the *Colidextribacter* genus was significantly higher in the S + Sty group compared to the LP, LP + Sty, and control groups, suggesting that the addition of the commercial probiotic to the drinking water significantly increased the relative abundance of *Colidextribacter* (*p* < 0.05). Compared to the control group, the relative abundance of the Lactobacillus genus was significantly higher in the LP, LP + Sty, and S + Sty groups, indicating that the addition of *L. plantarum* GX17 and the commercial probiotic to the drinking water significantly increased the relative abundance of Lactobacillus (*p* < 0.05).

## 4 Discussion

*S. typhimurium* is among the most perilous pathogens of infectious diseases, significantly impacting the poultry industry and causing substantial economic losses. Marcq et al. (2011) discovered that *Salmonella* infection could dramatically reduce the growth performance of broiler chickens. Many studies have also shown that probiotics can prevent weight losses caused by *Salmonella* infection in the livestock and poultry industries. Gong et al. (2015) found that the inclusion of *Bacillus subtilis* in the diet mitigated the growth retardation induced by *Salmonella* infection in broiler chickens. Similarly, Zhu et al. (2021) reported that when broiler chickens were orally administered *Bacillus subtilis* BC66 during an infection, both their weight loss and feed conversion ratio improved, suggesting the preventive and health-promoting effects of probiotics. However, there are fewer studies focused on the *Lactobacillus* strain group. In our research, broiler chickens fed with water contain either *L. plantarum* GX17 or a commercial probiotic. Both types of probiotics exhibited a significant improvement in the body weight of participants compared to the infected controls. Moreover, treated chickens might recover to a similar level as uninfected individuals. These results are consistent with other studies in the field, indicating that the addition of *L. plantarum* GX17 significantly mitigates weight loss caused by *S. typhimurium*. The thymus, spleen, and bursa of Fabricius have crucial immune functions during the development of chicks, and the immune organ index serves as a set of important indicators of immune functions. The bursa of Fabricius facilitates B cell differentiation and maturation (Masteller et al., 1997), while the spleen, which systemically regulates B cells and macrophages, plays a vital role in humoral immunity (Jia and Pamer, 2009). In this study, no statistically significant variations were observed in the sizes of the thymus, spleen, and bursa of Fabricius across the treatment groups, suggesting that infection and the following inflammation reactions of *S. typhimurium* were suppressed by the treatment of probiotics. Similar findings have been reported in experiments using compound probiotics for the prevention and treatment of *Salmonella* infection in broiler chickens (Neveling et al., 2020). Our results align with those studies: the *L. plantarum* GX17 and commercial probiotics used in this study did not lead to immune organ enlargement. Therefore, it can be inferred that the consumption of the probiotics used in this study may alleviate the symptoms of *Salmonella* infection, along with having a minor immunostimulatory effect on the immune organs of the chicks.

*Salmonella* can cause significant decline in broiler growth performance. The main reason for the decline in growth performance is the reduced feed intake of broilers due to mucosal damage, diarrhea and systemic infection (Vandeplas et al., 2009). Studies have found that *L. plantarum* significantly increased the levels of IL-10, while significantly reducing the levels of IL-4, IFN-γ, and TNF-α (Zhao et al., 2021). Another study showed that *L. plantarum* LS/07 can promote colon IL-10 production in inflammatory rats is used to treat inflammatory diseases (Štofilová et al., 2017), and studies have also shown that *L. plantarum* synergistically regulates M1 macrophage polarization to resist *S. typhimurium* infection (Duan et al., 2022). Therefore, the process by which *L. plantarum* GX17 alleviates the decline in growth performance may be related to its participation in activating the body’s immune defense function and alleviating the body’s inflammatory response.

Chicken meat is an important source of high-quality protein for humans, but it is also considered a major source of foodborne diseases, especially enteritis-causing *Salmonella* and typhoid-causing *Salmonella*. Therefore, reducing the contamination of live poultry with *S. typhimurium* can help mitigate the food safety risks faced by consumers. Studies have shown that the use of various probiotics in preventive treatments can control and reduce the colonization of *Salmonella* in the gastrointestinal tract of broiler chickens (Neveling et al., 2020). Additionally, research has demonstrated that treatment with probiotics based on lactic acid bacteria can significantly reduce the *Salmonella* count in chicks after infection (Menconi et al., 2011). Chen et al. (2017) found that adding *L. plantarum* Z01 to the diet significantly reduced the quantity of *S. typhimurium* in the cecum. In this study, adding *L. plantarum* GX17 and a commercial probiotic to the drinking water of newly hatched chicks significantly reduced the colonization of *S. typhimurium* in the tissues. Both *L. plantarum* GX17 and the commercial probiotic significantly decreased the content of *S. typhimurium* in the liver, spleen, and cecum tissues compared to the chicks without probiotic supplementation. The protective effect of probiotics may be attributed to the production of organic acids such as lactic acid, which has antimicrobial properties. The antimicrobial effects of organic acids are generally attributed to their ability to affect pH, intracellular osmotic pressure, cell membrane permeability, substance transport, cell signaling, energy metabolism, or biomacromolecule synthesis (Hauser et al., 2016). The anti-*Salmonella* mode of action of L-phenyllactic acid produced by *L. plantarum* is attributed to cell membrane disruption and genomic DNA-binding (Zhou et al., 2020). The antibacterial activity of *L. plantarum* ACA-DC287 against *S. typhimurium* SL1344 is due to the lactic acid it produces (Makras et al., 2006). Probiotics may also produce adhesion inhibitors like hydrogen peroxide and bacteriocins to reduce the chances of pathogens entering epithelial cells (Ohland and Macnaughton, 2010). Most lactic acid bacterial bacteriocins act by disrupting the cytoplasmic membrane through pore formation or by degrading the cell wall (Pérez-Ramos et al., 2021). BMP11, a new bacteriocin produced by *L. crustorum* against *Listeria monocytogenes*, has been shown to disrupt cell membrane integrity and increase membrane permeability (Cavicchioli et al., 2017). Bacteriocins produced by *L. plantarum* PTCC1745 significantly inhibited biofilm formation in *Acinetobacter baumannii*, reducing the expression of biofilm-related bap genes by 52% (Javadi et al., 2024). Therefore, it is speculated that the inhibition of *S. typhimurium* by *L. plantarum*m GX17 in this study may be caused by the production of antibacterial substances such as organic acids, hydrogen peroxide, and bacteriocins, but its specific destructive mechanism remains to be studied.

*Salmonella* enters the body through a gastrointestinal infection and spreads to other organs through the digestive tract or bloodstream (Beristain-Covarrubias et al., 2018). Studies have shown that *Salmonella* infection often leads to severe liver damage (Wu et al., 2014). In this study, the colonization level of *Salmonella* in the liver confirmed the protective effect of *L. plantarum* GX17 and the commercial probiotic on liver tissue. Feeding probiotics significantly reduced the content of *S. typhimurium* in the liver compared to chicks without probiotic supplementation. Furthermore, *L. plantarum* GX17 and the commercial probiotic alleviated the pathological damage caused by *S. typhimurium* in the liver. Similar results were also observed by Xing et al. (2021), which align with the findings of this study. The translocation of *S. typhimurium* is mainly due to the destruction of the intestinal barrier, which causes it to translocate to the spleen and liver. Studies have shown that the administration of *L. plantarum* LTC-113 can eliminate the increased permeability and bacterial translocation caused by *S. typhimurium*, which also reduces the damage to the spleen and liver of chicks (Wang et al., 2018). Therefore, the low bacterial load and less damage in the liver in this study may be due to the fact that *L. plantarum* GX17 alleviated the intestinal barrier damage caused by *S. typhimurium*, thereby reducing the number of *S. typhimurium* transferred from the intestine to the liver.

In both animal and human studies, the gut microbiota plays a significant role in maintaining various aspects of health, including immunity, metabolism, and neurobehavioral characteristics (Valdes et al., 2018). Research from the 1950s indicated that disrupting the microbiota increased susceptibility to infection (Bohnhoff et al., 1954). The advancement of sequencing technologies in the early 21st century enabled the analysis of microbial communities (Kamada et al., 2012; Barman et al., 2008; Stecher et al., 2007), and subsequent studies demonstrated that virulence factors can manipulate the microbial composition by targeting the host’s environment (Kamdar et al., 2016; Lopez et al., 2016; Winter et al., 2010). *S. typhimurium*, an important human pathogen, is often studied as a representative species (Ao et al., 2015; Majowicz et al., 2010). By inducing inflammation, *S. typhimurium* creates its own niche in the intestines, altering the composition of the gut microbiota and nutrient availability to favor its colonization and expansion (Ducarmon et al., 2019). The cecum of mice is known to have a high abundance of *S. typhimurium* (Carter and Collins, 1974), representing the largest microbial community in the body.

In this study, the 16S rRNA sequencing results revealed that the most common bacterial phyla in the cecum of broiler chickens were Firmicutes, Bacteroidetes, and Proteobacteria. Firmicutes, the predominant phylum, had the highest abundance (Wei et al., 2013). Certain members of Firmicutes can inhibit the growth of opportunistic pathogens, while others participate in the degradation of complex carbohydrates (Brown et al., 2012). Previous research has also shown that adding *Bacillus subtilis* to broiler chicken feed can regulate the intestinal immune system and gut microbiota, providing resistance against *Salmonella* infection (Hayashi et al., 2018). In this study, adding *L. plantarum* GX17 or a commercial probiotic to the drinking water could help resist the invasion of *S. typhimurium* on broiler chickens by modulating the abundance of *Anaerotruncus*, *Colidextribacter*, and *Lactobacillus*. *Clostridia*, which consist of various bacterial species, are closely associated with host inflammatory responses and serve as an important pathogen for producing botulinum toxins and causing fatal infections. It has been reported that an increasing level of *Anaerotruncus* is directly related to the elevation of pro-inflammatory cytokines associated with aging (Konkol et al., 2019). In mice fed a high-fat/high-cholesterol diet, an increase in *Anaerotruncus* abundance was found to be associated with hepatocellular carcinoma related to non-alcoholic fatty liver disease (NAFLD) (Zhang et al., 2020). Adding *L. plantarum* GX17 or a commercial probiotic to the drinking water restored the relative abundance of *Anaerotruncus* in the intestinal tract of broiler chickens infected with *S. typhimurium*, thereby alleviating the inflammatory response caused by the infection. Furthermore, adding the commercial probiotic to the drinking water significantly increased the relative abundance of *Colidextribacter* in the intestinal tract of *Salmonella*-infected broiler chickens. *Colidextribacter* promotes the production of guanosine, which helps reduce the secretion of inflammatory factors (Guo et al., 2021; Mager et al., 2020) and regulates systemic inflammatory responses while maintaining intestinal mucosal integrity (Liu et al., 2022). Increasing the proportion of *Lactobacillus* in the gut is desirable and beneficial. *Lactobacillus* is significantly positively correlated with the tight junction protein in the intestines and negatively correlated with inflammation and oxidative stress (Xia et al., 2020). Researchers have found that *L. plantarum* PS128 can regulate microbial communities, modulate inflammation, oxidative reactions, and protect gastrointestinal integrity, thereby affecting the physiological health of athletes, particularly their adaptability to exercise (Huang et al., 2020). Previous studies have shown that oral administration of probiotics can increase the relative abundance of *Lactobacillus* in the chicken gut (Yu et al., 2021). In this study, adding *L. plantarum* GX17 or a commercial probiotic to the drinking water significantly increased the relative abundance of *Lactobacillus* in the chicken gut. Compared to broiler chickens infected with *S. typhimurium* without probiotic supplementation, the addition of *L. plantarum* GX17 resulted in a significant increase in the relative abundance of *Lactobacillus* in the intestinal tract. Alternatively, this may be attributed to the proliferation of the administered probiotic strain, which produces molecules that stimulate the growth of *Lactobacillus* in the intestine (Ohashi et al., 2001; Takahashi et al., 2007). When a large number of probiotics enter the intestine, they adhere to the host’s epithelial cells, thereby eliminating the possibility of Salmonella colonization on the same substrate (Khochamit et al., 2020). In this study, colonization of *L. plantarum* GX17 into the intestine increased the number of lactic acid bacteria, and corresponding to the cecum bacterial load, the cecum *S. typhimurium* bacterial load significantly decreased after the addition of probiotics. Therefore, it was concluded that the competitive colonization of *L. plantarum* reduced the colonization amount of *Salmonella*, thus regulating the intestinal flora. Improves the immune system and maintains the function of the intestinal barrier.

## 5 Conclusion

In general, we demonstrated that *L. plantarum* GX17 can prevent the damage caused by *S. typhimurium* infection by modulating the gut microbiota. In this study, *L. plantarum* GX17 showed a preventive and protective effect in the *S. typhimurium* infection model. Our study represents a potential anti-infection strategy that uses probiotics to prevent and resist pathogen infections, possibly by modulating the gut microbiota of the host.

## Data Availability

The datasets presented in this study can be found in online repositories. The names of the repository/repositories and accession number(s) can be found at: https://www.ncbi.nlm.nih.gov/, PRJNA943474.

## Supplementary material

SUPPLEMENTARY FIGURE S1 Comprehensive analysis of microbial diversity. (A) Good’s coverage analysis: this panel depicts the sequencing coverage of the sample data, indicating the relationship between the sequencing depth and the estimated species richness. (B) Dilution curve: the dilution curve illustrates the species richness as a function of the number of sequences sampled. This curve is used to estimate the total number of species present in the sample and to determine if the sampling effort is sufficient to capture the majority of the species diversity. (C) Accumulation curve: the accumulation curve shows the increase in the number of observed species with the addition of more samples or sequences. It is a graphical representation of species richness over the sampling effort, helping to identify if the sampling is exhaustive or if more samples are needed to achieve a comprehensive understanding of the microbial community’s diversity. Control group: chicks were orally administered PBS on the 8th and 9th days. LP group: chicks were orally administered *L. plantarum* from the 1st to the 7th day and given PBS on the 8th and 9th days. LP + Sty group: chicks were orally administered *L. plantarum* from the 1st to the 7th day and were given *S. typhimurium* on the 8th and 9th days. S + Sty group: chicks were orally administered a commercial probiotic from the 1st to the 7th day and given PBS on the 8th and 9th days. Sty group: chicks were orally administered *S. typhimurium* on the 8th and 9th days.
