# Supplementary figures and images for: Enhancing resistance to Salmonella typhimurium in yellow-feathered broilers: a study of a strain of Lactiplantibacillus plantarum as probiotic feed additives
Source: Front Microbiol. 2024 Nov 20;15:1450690. doi: 10.3389/fmicb.2024.1450690 (PMC11615061; doi:10.3389/fmicb.2024.1450690)

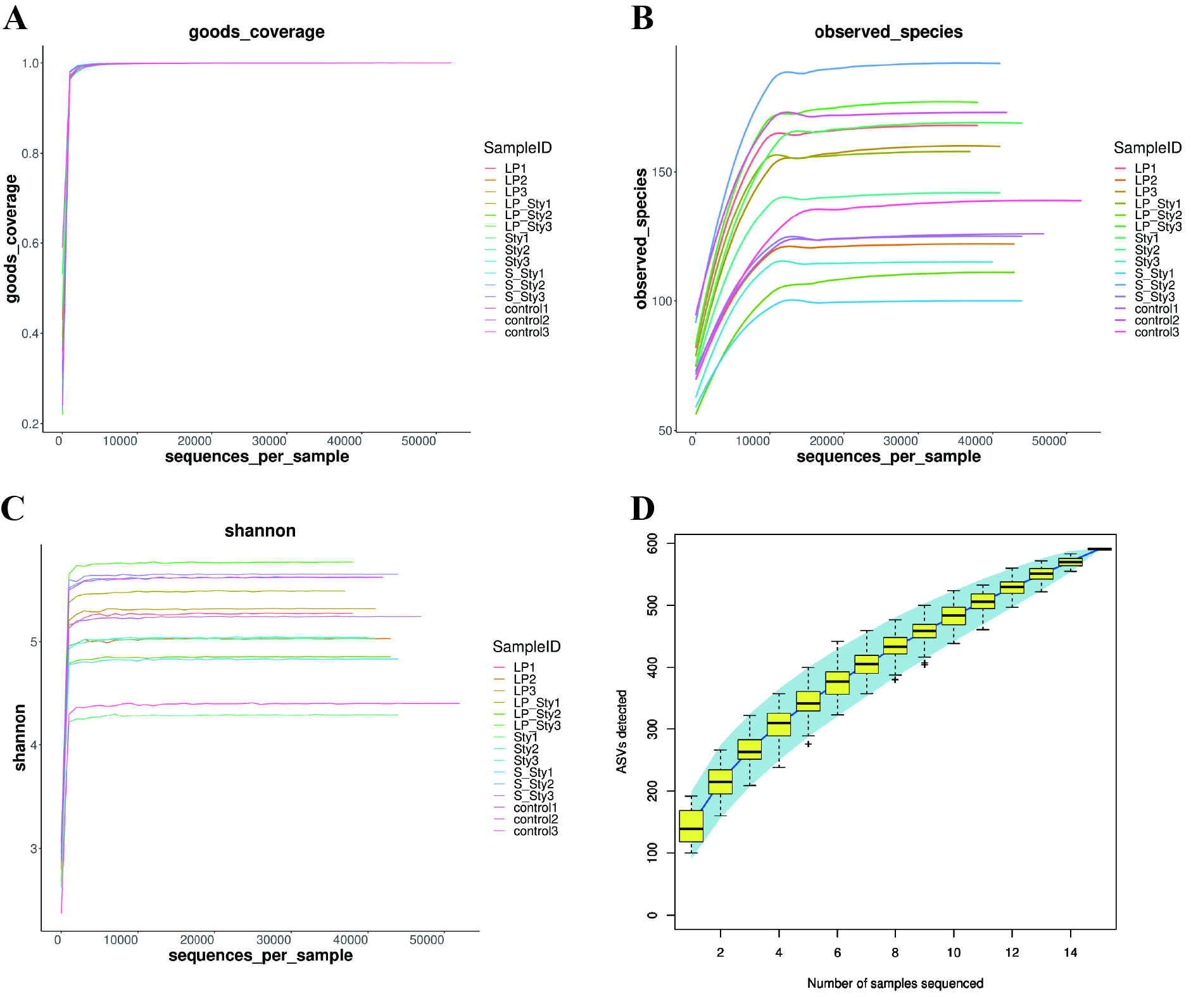

Supplement: SUPPLEMENTARY FIGURE S1 — Comprehensive analysis of microbial diversity. (A) Good’s coverage analysis: this panel depicts the sequencing coverage of the sample data, indicating the relationship between the sequencing depth and the estimated species richness. (B) Dilution curve: the dilution curve illustrates the species richness as a function of the number of sequences sampled. This curve is used to estimate the total number of species present in the sample and to determine if the sampling effort is sufficient to capture the majority of the species diversity. (C) Accumulation curve: the accumulation curve shows the increase in the number of observed species with the addition of more samples or sequences. It is a graphical representation of species richness over the sampling effort, helping to identify if the sampling is exhaustive or if more samples are needed to achieve a comprehensive understanding of the microbial community’s diversity. Control group: chicks were orally administered PBS on the 8th and 9th days. LP group: chicks were orally administered L. plantarum from the 1st to the 7th day and given PBS on the 8th and 9th days. LP + Sty group: chicks were orally administered L. plantarum from the 1st to the 7th day and were given S. typhimurium on the 8th and 9th days. S + Sty group: chicks were orally administered a commercial probiotic from the 1st to the 7th day and given PBS on the 8th and 9th days. Sty group: chicks were orally administered S. typhimurium on the 8th and 9th days. [file Image_1.TIF]
